# Herpes Zoster and Venous Thromboembolism Following Upadacitinib Treatment for Severe Atopic Dermatitis

**DOI:** 10.7759/cureus.71007

**Published:** 2024-10-07

**Authors:** Kawaiola Cael Aoki, Colin Burnette, Simona Bartos

**Affiliations:** 1 Dr. Kiran C. Patel College of Osteopathic Medicine, Nova Southeastern University, Fort Lauderdale, USA; 2 Dermatology, Imperial Dermatology, Hollywood, USA

**Keywords:** atopic dermatitis, biologics, eczema, herpes zoster, jak inhibitor, shingles, upadacitinib, venous thromboembolism

## Abstract

New medications targeting the Janus kinase (JAK)-signal transducer and activator of transcription (STAT) pathway have been developed through emerging biologics research. However, due to documented adverse effects, including herpes zoster (HZ) and venous thromboembolism (VTE), an extensive patient workup and counseling are necessary before prescribing. We present the case of an 81-year-old patient with severe atopic dermatitis on upadacitinib, a selective JAK1 inhibitor, who developed HZ and VTE, requiring hospitalization. This study emphasizes the need for further research, continuous monitoring, and risk management for HZ and VTE in patients undergoing upadacitinib treatment, especially in high-risk populations.

## Introduction

The Janus kinase (JAK)-signal transducer and activator of transcription (STAT) pathway plays a crucial role in the development of immune-related conditions, such as rheumatoid arthritis (RA), psoriatic arthritis, inflammatory bowel disease, and atopic dermatitis (AD). To target JAK-STAT proteins, small-molecule inhibitors (JAKi) competitively bind to the ATP-binding site, thereby disrupting signal transduction and impairing cytokine signaling [1]. Upadacitinib (UPA) is a once-daily oral medication that selectively inhibits JAK1, with limited influence on JAK2, JAK3, and tyrosine kinase two [1-3]. Upadacitinib inhibits pro-inflammatory signaling pathways, including IL-4, IL-5, IL-13, and IL-31, which significantly contribute to AD [1].

Herpes zoster (HZ), commonly known as shingles, is caused by the reactivation of the varicella-zoster virus (VZV) after its initial infection and establishment of latency in the dorsal root ganglia or cranial nerves [4]. Monocytes and natural killer cells produce cytokines-IFNs, TNF, and IL-12, which induce the maturation of VZV-specific T cells, crucial for resolving the primary infection and controlling reactivations [5]; however, when downregulated, it can facilitate virus replication, leading to the development of shingles [6]. JAKi have been proven to increase the risk of HZ, as these medications can affect the typical immune response. Furthermore, different JAKi produce similar immunogenic effects, even when selectively targeting JAKs [4,7].

## Case presentation

**Figure 1:**
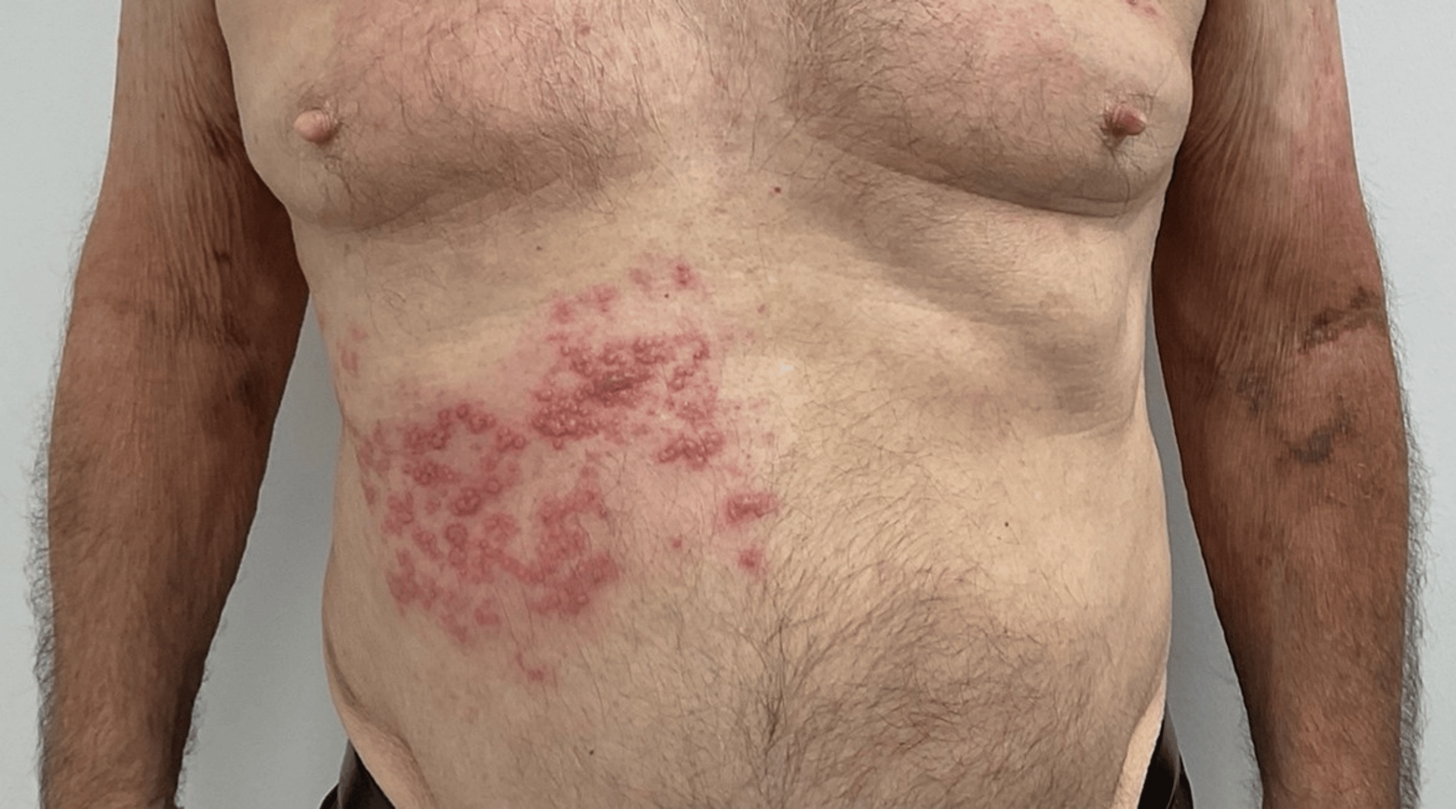
Herpetiform papulovesicules in a dermatomal distribution, anterior view.

**Figure 2:**
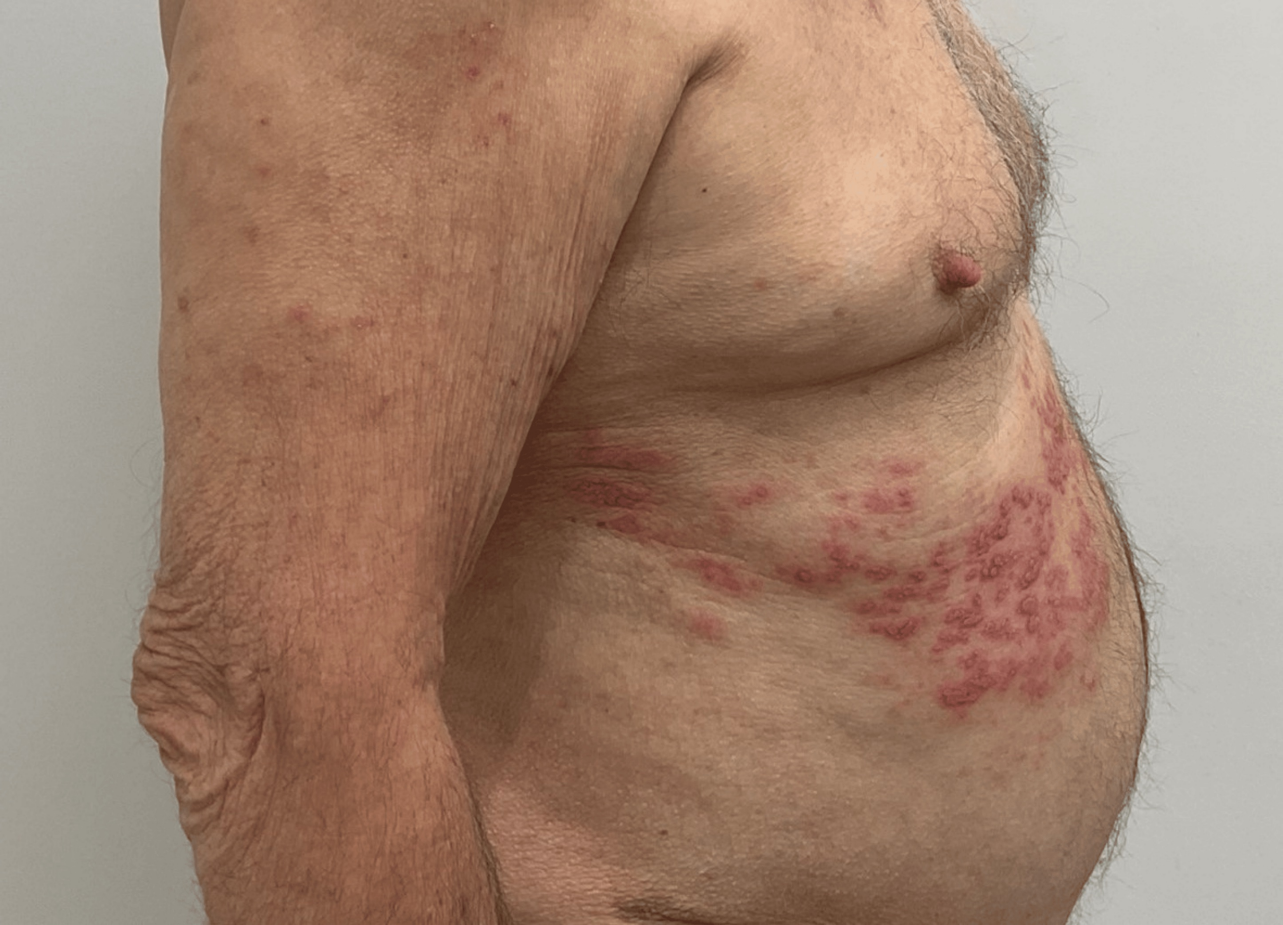
Herpetiform papulovesicules in a dermatomal distribution, lateral view.

An 81-year-old male patient who has been undergoing treatment for AD for the past four months has a history of hypertension, congestive heart failure, hypercholesterolemia, basal cell carcinoma, and squamous cell carcinoma. Despite multiple therapies, including dupilumab 300 mg/2 mL injection, mupirocin 2% ointment, ivermectin 1% cream, and triamcinolone 40 mg/mL injection, his AD remained nonresponsive. His AD was eventually managed with a combination of triamcinolone 0.1% ointment and UPA 15-mg extended-release tablets, which he continued to use for three months with success. The patient was informed of the associated risks and advised to receive the shingles vaccine but did not follow the recommendation. Baseline laboratory results were reviewed, and written informed consent was obtained. He had no personal or family history of clotting disorders, so no further workup was deemed necessary. The patient was advised to receive the shingles vaccine but did not follow the recommendation. At this visit, he presented with new painful, clustered vesicles on an erythematous base arranged in a dermatomal distribution under his right chest (Figures 1, 2). After being diagnosed with HZ, the patient's UPA was promptly discontinued. The patient was prescribed valacyclovir 1000 mg TID for seven days, and within a week, the HZ had resolved.

**Figure 3:**
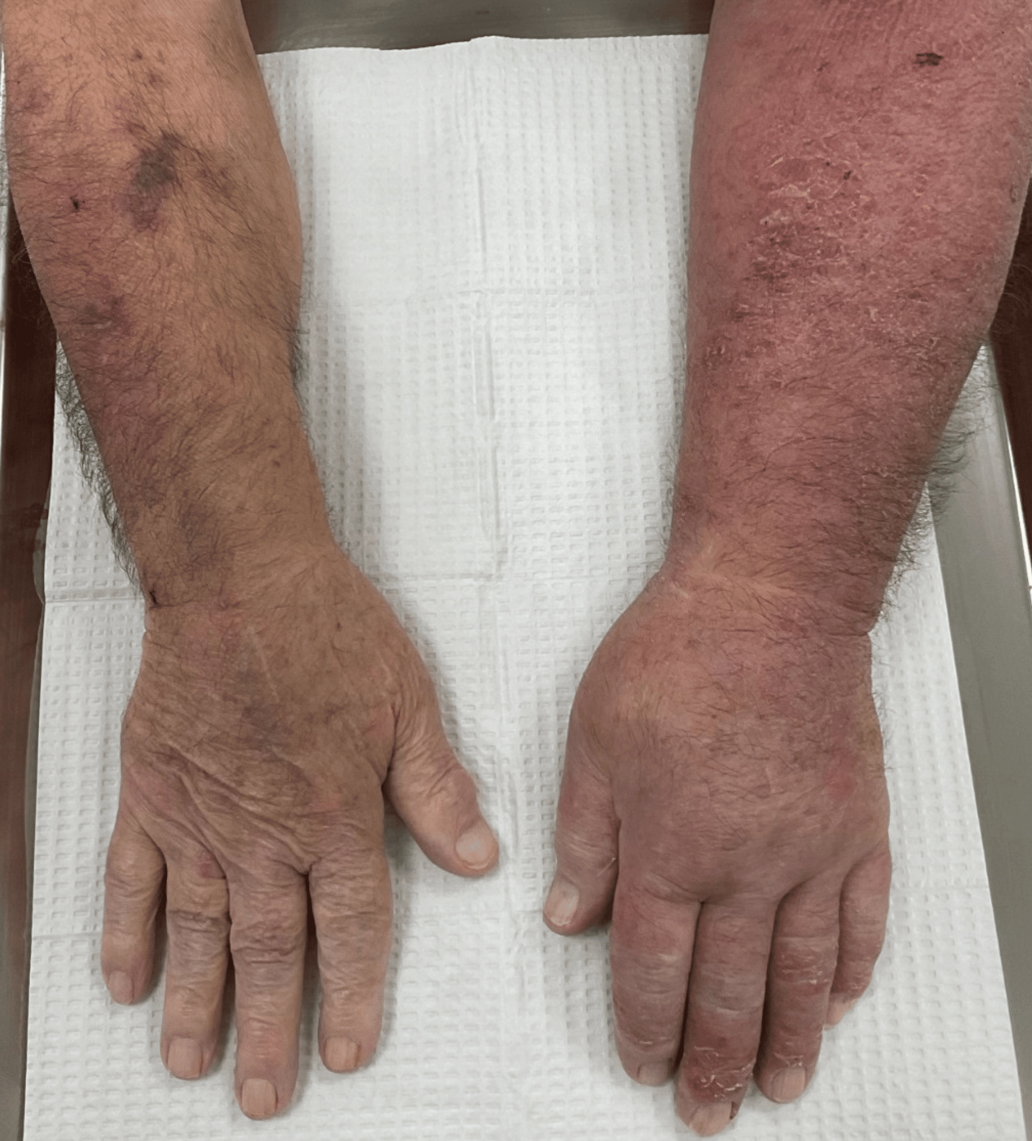
Edema and erythema of left upper extremity secondary to deep vein thrombosis.

Approximately one week later, the patient reported experiencing redness and swelling in the left upper extremity (Figure 3). Upon conducting an ultrasound, the left cephalic and left basilic vein revealed a deep vein thrombosis partially occluding the vasculature, and the patient was urgently referred to the emergency department. After receiving prompt medical attention, which included the administration of rivaroxaban, the patient demonstrated a complete recovery and was ultimately discharged in a satisfactory condition.

**Figure 4:**
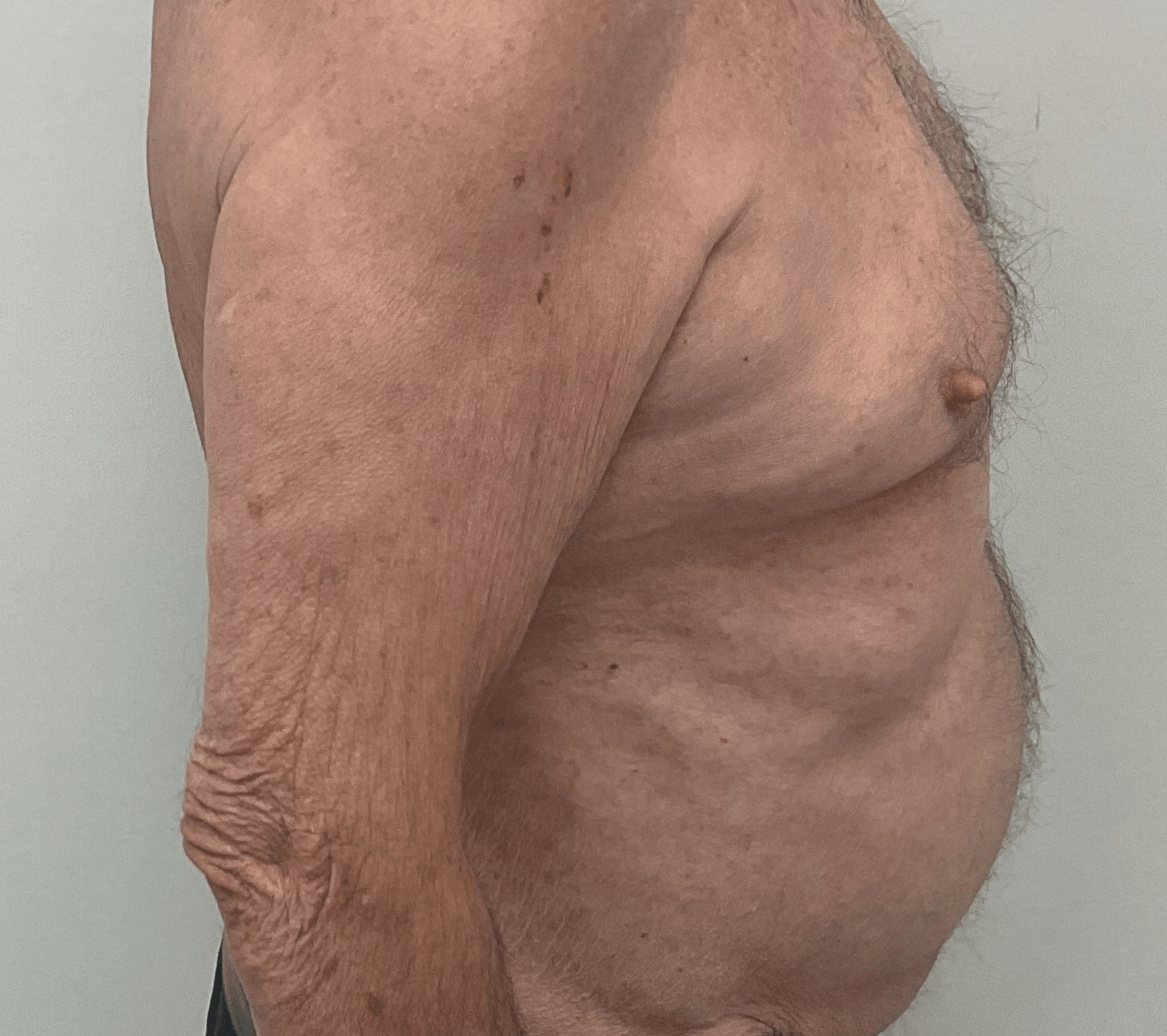
Shingles and atopic dermatitis resolution, anterior view.

**Figure 5:**
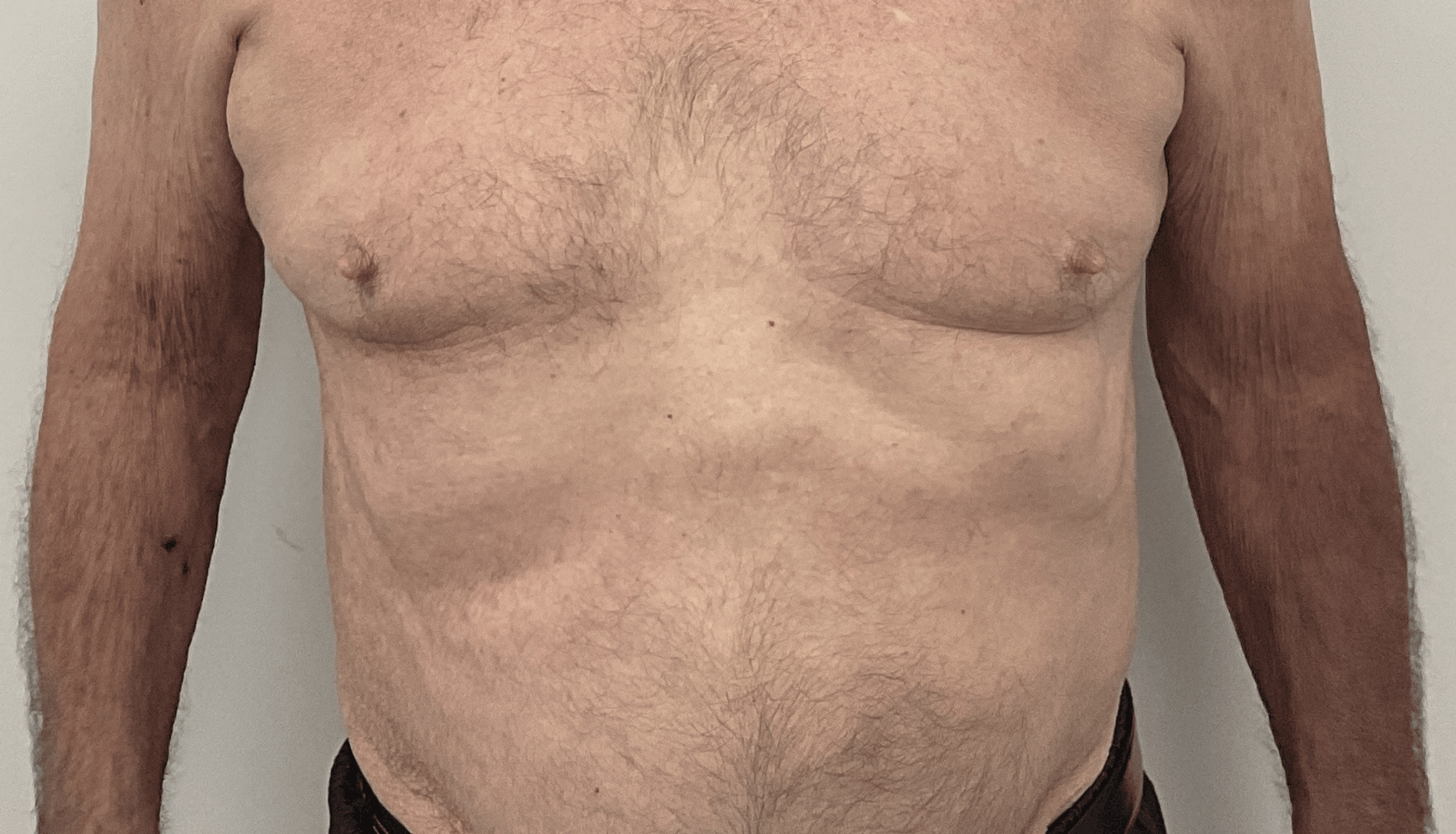
Shingles and atopic dermatitis resolution, anterior view.

Given that his AD was resistant to dupilumab and resembled a psoriasiform eruption, a trial of risankizumab 150 mg was initiated for its potential efficacy in psoriatic conditions and off-label use for AD. However, the treatment was quickly discontinued due to the lack of effectiveness, and the patient was subsequently switched to a one-month series of tralokinumab followed by a one-month course of deucravacitinib, both of which failed to improve symptoms. Clinical improvement was eventually achieved through a regimen of 25 mg twice-daily oral cyclosporine, once-daily 1.5% topical minocycline 0.1% and tacrolimus ointment for the face, and 0.1% triamcinolone acetonide ointment for the body (Figures 4, 5).

At a follow-up visit conducted three months after the change in treatment, the patient remained symptom-free, with no recurrence of either atopic dermatitis or herpes zoster. In addition, the patient showed no further signs of thromboembolic events. The patient was treated exclusively at Imperial Dermatology, with visiting medical students from different institutions observing his care during their rotations. The patient continues to be regularly monitored at Imperial Dermatology to ensure ongoing stability.

## Discussion

Although JAK inhibitors (JAKi) come with a boxed warning regarding the risk of serious infections, including herpes zoster (HZ), the relative incidence of HZ in AD patients remains unclear due to limited research on these novel drugs. In a phase III Japanese RCT (n = 264), the occurrence rate of HZ infection was higher in the 30 mg group (14.7%) compared to the 15 mg group (7.2%) and placebo. Most HZ events were mild to moderate, affecting only one dermatome, and none involved the central nervous system [8]. In a single-center retrospective chart review of AD patients aged 50 years or older (n = 7), no HZ events were observed when a prophylactic shingles vaccine was administered prior to tofacitinib or UPA adherence [9].

Moreover, data from RA studies can provide additional insight: Two meta-analyses revealed that a small percentage of patients experienced severe HZ events, which was higher in UPA than in other treatment groups [2,3]. In a study of 3,834 patients treated with UPA, the rates of HZ were 3.4% and 6.3% at 15 mg and 30 mg doses, respectively [3]. In another analysis of 5,306 patients, 6.4% and 12.5% of patients in the 15 and 30 mg treatment groups had HZ. In the UPA 15 mg group, 0.3% of patients had severe HZ events; in the UPA 30 mg group, the rate was 1.2% [2]. In both studies, older patients and those in Asian populations had notable elevations in HZ rates [2,3].

Venous thromboembolism (VTE) is another reported complication among all JAKi, requiring immediate drug discontinuation. JAKi demonstrated a nearly twofold higher VTE occurrence rate (1.13%) than all other biologics (0.61%) after one year [10]. However, UPA showed no statistically significant differences in VTE risk in either a meta-analysis of six phase III RCTs of RA patients (n = 3,209) [11], a retrospective study of two phase III RCTs of AD patients (n = 1,609) [12], or 48-week observational study of 146 AD patients [13]. Notably, individuals who experienced VTE exhibited increased cardiovascular risk before treatment: prior thromboembolic event, hypertension, smoking history, or age exceeding 65 [12,13]. Before prescribing UPA treatment for AD, the patient’s medical history should be considered to assess these risk factors.

When evaluating the risk associated with JAKi, the severity and seriousness of adverse events, such as HZ and VTE, are crucial considerations. Severity refers to the intensity of the event. In this case, the patient experienced mild HZ, which affected one dermatome and resolved with antiviral treatment. Conversely, the VTE was more severe, requiring hospitalization and urgent anticoagulant therapy. Seriousness, on the other hand, involves the potential consequences, including hospitalization and life-threatening conditions. The VTE in this patient was classified as a serious adverse event (SAE), particularly given the patient’s advanced age and underlying conditions.

The dechallenge of discontinuing upadacitinib after the onset of HZ and VTE resolved both conditions, suggesting a likely link between the medication and the adverse events. Although rechallenge, or reintroducing the drug to confirm this causality, was not attempted due to the seriousness of the events, it remains a standard method for establishing definitive drug-related causes. However, in cases where adverse events pose a significant risk, rechallenge is typically avoided.

Assessing causality is key to understanding whether a medication is responsible for adverse events. The temporal association between upadacitinib use, the onset of HZ and VTE, and the resolution following discontinuation supports a probable causal relationship. Although confounding factors such as the patient’s age and comorbidities may have contributed to these events, the clear timing and improvement with dechallenge indicate that upadacitinib played a significant role.

While severe infections like herpes zoster can theoretically contribute to a pro-thrombotic state, the patient's clinical presentation and timing of events suggest that the deep vein thrombosis might be associated with multiple factors, including using upadacitinib. Further research is warranted to investigate the impact of JAKi on AD patients, including their association with the development of VTE and HZ. Efforts should aim to enhance understanding of adverse events in JAKi use, including predictors and long-term outcomes, for safety optimization in diverse population groups based on age, ethnicity, and comorbidities.

## Conclusions

JAKi have become increasingly important in managing conditions like RA and AD but pose potential risks despite their generally favorable safety profile. While the exact mechanisms behind UPA-related HZ and VTE are not fully understood, current evidence suggests a complex interaction between immune modulation and thrombotic pathways. The case presented underscores two of the serious complications associated with UPA therapy. Vigilant monitoring and risk management strategies are necessary to mitigate these risks, and clinicians must weigh them against the potential benefits of JAKi, especially in high-risk patient populations. Furthermore, the differences in HZ and VTE rates among various populations highlight the significance of individualized risk evaluation and counseling for patients. While the exact contribution of herpes zoster to the development of DVT remains unclear, the potential risk associated with upadacitinib, in conjunction with other factors, warrants further investigation. Clinicians should consider the multifactorial nature of thromboembolic events when managing patients on JAKi.
